# Glutamatergic dysfunction in resistant obsessive-compulsive disorder: An auditory mismatch negativity study

**DOI:** 10.1192/j.eurpsy.2021.1118

**Published:** 2021-08-13

**Authors:** F. Syed, N. Thatikonda, S. Arumugham, J. Narayanaswamy, V. Ganesan, Y.J. Reddy

**Affiliations:** 1 Department Of Clinical Neurosciences, National Institute of Mental Health and Neurosciences, Bengaluru, India; 2 Psychiatry, NIMHANS, Bengaluru, India

**Keywords:** Obsessive-Compulsive disorder, glutamate in OCD, mismatch negativity, resistant OCD

## Abstract

**Introduction:**

Obsessive-compulsive disorder (OCD) patients with poor response to serotonin reuptake inhibitors (SRIs) may have dysfunction involving other neurotransmitters, including glutamate. Mismatch negativity (MMN), an event-related potential dependent on glutamatergic functioning, has not been studied in the adult OCD population and SRI non-responders.

**Objectives:**

To compare the amplitude of MMN between OCD subjects who have responded(R) and not responded(NR) to SRIs, with healthy volunteers(HV).

**Methods:**

MMN was measured in 15 OCD subjects fulfilling DSM-IV criteria (8 non-responders and 7 responders) and 22 healthy volunteers. Auditory MMN was measured using a multi-feature paradigm consisting of two variants each in frequency, duration, and intensity domains. EEG was recorded using 64 channel electrodes at 1000Hz. Epochs of 700 ms were extracted for each stimulus. MMN was evaluated as peak difference between the deviant and standard stimulus. MMN amplitudes at Fz were used for comparison between the groups using Kruskal-Wallis test followed by posthoc analysis, with significance set at p<0.05.

**Results:**

There was no significant difference in age/gender distribution between the three groups and duration of illness between the two OCD groups. There was a significant difference in MMN amplitude of a frequency deviant between the three groups (H=7.312,P=0.026). Post-hoc pairwise analyses revealed a significant reduction in MMN amplitude in NRs as compared to the HV group (H=10.9,P=0.04).

**Conclusions:**

The results are suggestive of glutamatergic dysfunction in OCD subjects with poor response to SRIs. The findings have to be replicated in larger samples employing other paradigms to evaluate glutamatergic functioning and have future potential in understanding treatment response to SRIs.

